# Draft genome sequence of a highly proteolytic *Staphylococcus aureus* USA300 isolate from human urine

**DOI:** 10.1128/MRA.00679-23

**Published:** 2023-11-07

**Authors:** Jessica K. Jackson, Sarah J. Kennedy, Emily A. Felton, Eleonora Cella, Amorce Lima, Deanna Becker, Suzane Silbert, Kami Kim, Taj Azarian, Lindsey N. Shaw

**Affiliations:** 1 Department of Molecular Biosciences, University of South Florida, Tampa, Florida, USA; 2 Burnett School of Biomedical Sciences, University of Central Florida, Orlando, Florida, USA; 3 Esoteric Testing/R&D Laboratory, Tampa General Hospital, Tampa, Florida, USA; 4 Division of Infectious Disease and Internal Medicine, University of South Florida, Tampa, Florida, USA; 5 Center for Global Health Infectious Diseases Research, University of South Florida, Tampa, Florida, USA; 6 Global Emerging Diseases Institute, Tampa General Hospital, Tampa, Florida, USA; Rochester Institute of Technology, Rochester, New York, USA

**Keywords:** *Staphylococcus aureus*, USA300, proteases, clinical isolate

## Abstract

The secreted proteases of *Staphylococcus aureus* have been shown to be critical during infection. Here, we present the draft genome sequence of *S. aureus* TGH337, a hyper-proteolytic USA300 strain isolated from human urine.

## ANNOUNCEMENT

The 10 secreted proteases of *Staphylococcus aureus* cleave both host proteins and self-derived virulence factors (1–7), and as a result, the abundance of these proteases has been shown to greatly influence disease outcome (8–10). Therefore, observation regarding the proteolytic variation present in clinical strains of *S. aureus* can be highly informative regarding the virulence mechanisms used by this important human pathogen.

**Fig 1:**
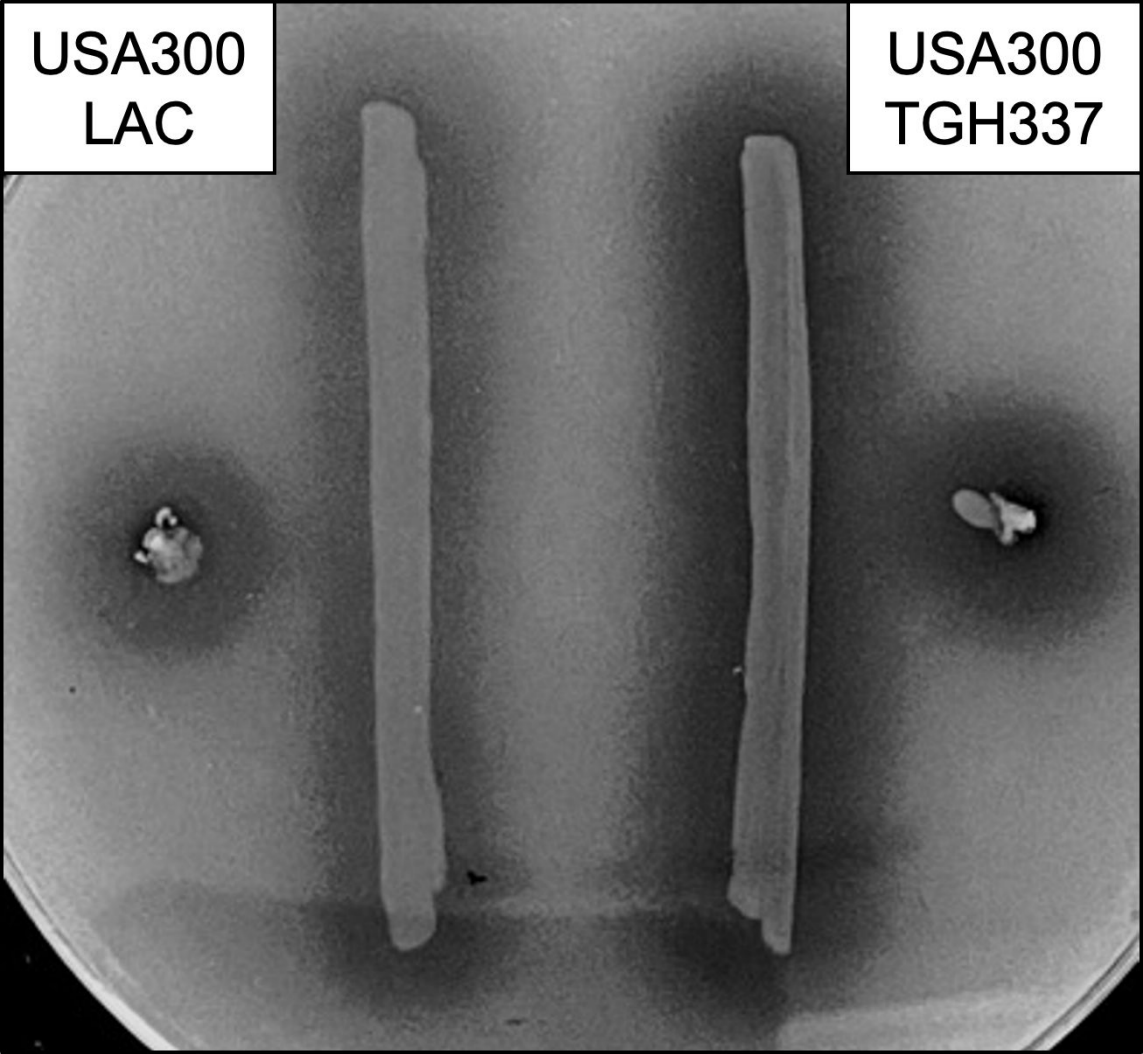
Hyper-proteolysis of TGH337 observed on casein agar plates in comparison to LAC. Casein agar plates [TSA with 5% (wt/vol) dry milk] were inoculated with LAC and TGH337 and incubated statically at 37°C for 18 h. After incubation, they were placed at 4°C for 4 h and then imaged on a Bio-Rad ChemiDoc Imaging System. The increased proteolysis of TGH337 can be observed by the increased zone of clearing around TGH337 in comparison to that around LAC.

USA300 pulse-field type strains are among the most prevalent *S. aureus* isolates in the United States, causing myriad community- and hospital-acquired infections (11–13). They are characterized by an overproduction of classical virulence factors compared to strains from other clonal complexes (14–16). Despite this observation, isolate TGH337 displays a unique hyper-proteolytic phenotype (Fig. 1) even for USA300 strains. Understanding the mechanism behind, and the fitness cost associated with, this novel phenotype will provide important insight into the regulation of the infectious process for this important human pathogen.

TGH337 was collected via mid-stream clean catch at Tampa General Hospital in Tampa, Florida. This urine sample was then plated on both blood agar and MacConkey agar. TGH337 was taken from the blood agar plate and subcultured on blood agar to ensure purity. Bacterial DNA extraction and whole genome sequencing were carried out as previously described (17). Briefly, TGH337 was grown overnight (37°C, 200  rpm) in tryptic soy broth (BD, Heidelberg, Germany), and then treated with 0.1 mg/mL lysostaphin. Genomic DNA was extracted using the Qiagen DNeasy Blood & Tissue kit with 50 mg/mL lysozyme added to the lysis buffer. DNA quality and quantity were assessed using a Qubit 4 Fluorometer. An Illumina MiSeq instrument with a Nextera Flex library preparation kit and V3 flow cell chemistry produced 173.2 Mbp (694,820) of paired-end 2 × 250 bp reads (>61× idealized coverage). The raw reads were trimmed via Trimmomatic v0.39 (18) with SLIDINGWINDOW:10:20 MINLEN:31 TRAILING:2 settings (19). Trimming quality checks were performed with FastQC v0.11.9 and MultiQC v1.12 (20). The reads were then *de novo* assembled with SPAdes v3.15.4 default settings (21) and assembly was checked for quality via Quast v5.0.2 (22).

The TGH337 genome assembly was generated from 677,633 reads to yield 31 contigs with a total length of 2,837,418 bp and mean coverage of 58× depth (*N_50_* 379,289 bp; largest contig, 881 Kbp). The G + C content is 32.66%, and PHASTER analysis (23) detected two intact and two incomplete prophage regions in TGH337.

## Data Availability

The full sequencing project’s raw reads are available in SRA study SRP349141; the TGH337 BioProject accession is PRJNA785927, with BioSample SAMN23599515 and SRA accession number SRS11212855. This Whole Genome Shotgun project has been deposited at DDBJ/ENA/GenBank under the accession JAULTO000000000. The version described in this paper is version JAULTO010000000.

## References

[1] Ohbayashi T, Irie A, Murakami Y, Nowak M, Potempa J, Nishimura Y, Shinohara M, Imamura T. 2011. Degradation of fibrinogen and collagen by staphopains, cysteine proteases released from Staphylococcus aureus. Microbiology 157:786–792. doi:10.1099/mic.0.044503-0 21081759

[2] Potempa J, Dubin A, Korzus G, Travis J. 1988. Degradation of Elastin by a Cysteine Proteinase from Staphylococcus aureus. J Biol Chem 263:2664–2667. doi:10.1016/S0021-9258(18)69118-5 3422637

[3] Paharik AE, Salgado-Pabon W, Meyerholz DK, White MJ, Schlievert PM, Horswill AR. 2016. The spl serine proteases modulate Staphylococcus aureus protein production and virulence in a rabbit model of pneumonia. mSphere 1:e00208-16. doi:10.1128/mSphere.00208-16 27747296 PMC5061998

[4] Laarman AJ, Ruyken M, Malone CL, van Strijp JAG, Horswill AR, Rooijakkers SHM. 2011. Staphylococcus aureus metalloprotease aureolysin cleaves complement C3 to mediate immune evasion. J Immunol 186:6445–6453. doi:10.4049/jimmunol.1002948 21502375

[5] McGavin MJ, Zahradka C, Rice K, Scott JE. 1997. Modification of the Staphylococcus aureus fibronectin binding phenotype by V8 protease. Infect Immun 65:2621–2628. doi:10.1128/iai.65.7.2621-2628.1997 9199429 PMC175371

[6] Karlsson A, Saravia-Otten P, Tegmark K, Morfeldt E, Arvidson S. 2001. Decreased amounts of cell wall-associated protein A and fibronectin-binding proteins in Staphylococcus aureus sarA mutants due to up-regulation of extracellular proteases. Infect Immun 69:4742–4748. doi:10.1128/IAI.69.8.4742-4748.2001 11447146 PMC98560

[7] McAleese FM, Walsh EJ, Sieprawska M, Potempa J, Foster TJ. 2001. Loss of clumping factor B fibrinogen binding activity by Staphylococcus aureus involves cessation of transcription, shedding and cleavage by metalloprotease. J Biol Chem 276:29969–29978. doi:10.1074/jbc.M102389200 11399757

[8] Gimza BD, Jackson JK, Frey AM, Budney BG, Chaput D, Rizzo DN, Shaw LN. 2021. Unraveling the impact of secreted proteases on hypervirulence in Staphylococcus aureus. mBio 12:e03288-20. doi:10.1007/978-1-0716-1550-8_15 33622717 PMC8545110

[9] Kolar SL, Ibarra JA, Rivera FE, Mootz JM, Davenport JE, Stevens SM, Horswill AR, Shaw LN. 2013. Extracellular proteases are key mediators of Staphylococcus aureus virulence via the global modulation of virulence-determinant stability. Microbiologyopen 2:18–34. doi:10.1002/mbo3.55 23233325 PMC3584211

[10] Zielinska AK, Beenken KE, Mrak LN, Spencer HJ, Post GR, Skinner RA, Tackett AJ, Horswill AR, Smeltzer MS. 2012. sarA-mediated repression of protease production plays a key role in the pathogenesis of Staphylococcus aureus USA300 isolates. Mol Microbiol 86:1183–1196. doi:10.1111/mmi.12048 23075270 PMC3508076

[11] Linz MS, Mattappallil A, Finkel D, Parker D. 2023. Clinical impact of Staphylococcus aureus skin and soft tissue infections. Antibiotics (Basel) 12:557. doi:10.3390/antibiotics12030557 36978425 PMC10044708

[12] David MZ, Daum RS. 2017. Treatment of Staphylococcus aureus infections. Curr Top Microbiol Immunol 409:325–383. doi:10.1007/82_2017_42 28900682

[13] Uhlemann A-C, Otto M, Lowy FD, DeLeo FR. 2014. Evolution of community- and healthcare-associated methicillin-resistant Staphylococcus aureus. Infect Genet Evol 21:563–574. doi:10.1016/j.meegid.2013.04.030 23648426 PMC3884050

[14] DeLeo FR, Otto M, Kreiswirth BN, Chambers HF. 2010. Community-associated meticillin-resistant Staphylococcus aureus. Lancet 375:1557–1568. doi:10.1016/S0140-6736(09)61999-1 20206987 PMC3511788

[15] Li M, Diep BA, Villaruz AE, Braughton KR, Jiang X, DeLeo FR, Chambers HF, Lu Y, Otto M. 2009. Evolution of virulence in epidemic community-associated methicillin-resistant Staphylococcus aureus. Proc Natl Acad Sci USA 106:5883–5888. doi:10.1073/pnas.0900743106 19293374 PMC2667066

[16] Otto M. 2013. Community-associated MRSA: what makes them special? Int J Med Microbiol 303:324–330. doi:10.1016/j.ijmm.2013.02.007 23517691 PMC3729626

[17] Cella E, Sutcliffe CG, Tso C, Paul E, Ritchie N, Colelay J, Denny E, Grant LR, Weatherholtz RC, Hammitt LL, Azarian T. 2022. Carriage prevalence and genomic epidemiology of Staphylococcus aureus among native American children and adults in the Southwestern USA. Microb Genom 8:mgen000806. doi:10.1099/mgen.0.000806 35551692 PMC9465076

[18] Bolger AM, Lohse M, Usadel B. 2014. Trimmomatic: a flexible trimmer for Illumina sequence data. Bioinformatics 30:2114–2120. doi:10.1093/bioinformatics/btu170 24695404 PMC4103590

[19] Chen C, Khaleel SS, Huang H, Wu CH. 2014. Software for pre-processing Illumina next-generation sequencing short read sequences. Source Code Biol Med 9:8. doi:10.1186/1751-0473-9-8 24955109 PMC4064128

[20] Ewels P, Magnusson M, Lundin S, Käller M. 2016. Multiqc: summarize analysis results for multiple tools and samples in a single report. Bioinformatics 32:3047–3048. doi:10.1093/bioinformatics/btw354 27312411 PMC5039924

[21] Prjibelski A, Antipov D, Meleshko D, Lapidus A, Korobeynikov A. 2020. Using spades de novo assembler. Curr Protoc Bioinformatics 70:e102. doi:10.1002/cpbi.102 32559359

[22] Mikheenko A, Prjibelski A, Saveliev V, Antipov D, Gurevich A. 2018. Versatile genome assembly evaluation with QUAST-LG. Bioinformatics 34:i142–i150. doi:10.1093/bioinformatics/bty266 29949969 PMC6022658

[23] Arndt D, Grant JR, Marcu A, Sajed T, Pon A, Liang Y, Wishart DS. 2016. PHASTER: a better, faster version of the PHAST Phage search tool. Nucleic Acids Res 44:W16–W21. doi:10.1093/nar/gkw387 27141966 PMC4987931

